# Supplementary figures and images for: Characteristics of Subtype and Molecular Transmission Networks among Newly Diagnosed HIV-1 Infections in Patients Residing in Taiyuan City, Shanxi Province, China, from 2021 to 2023
Source: Viruses. 2024 Jul 22;16(7):1174. doi: 10.3390/v16071174 (PMC11281631; doi:10.3390/v16071174)

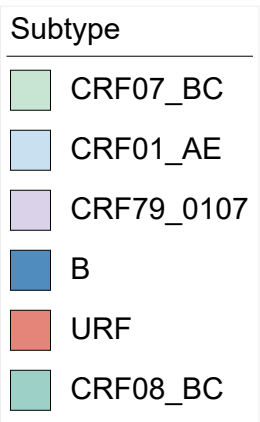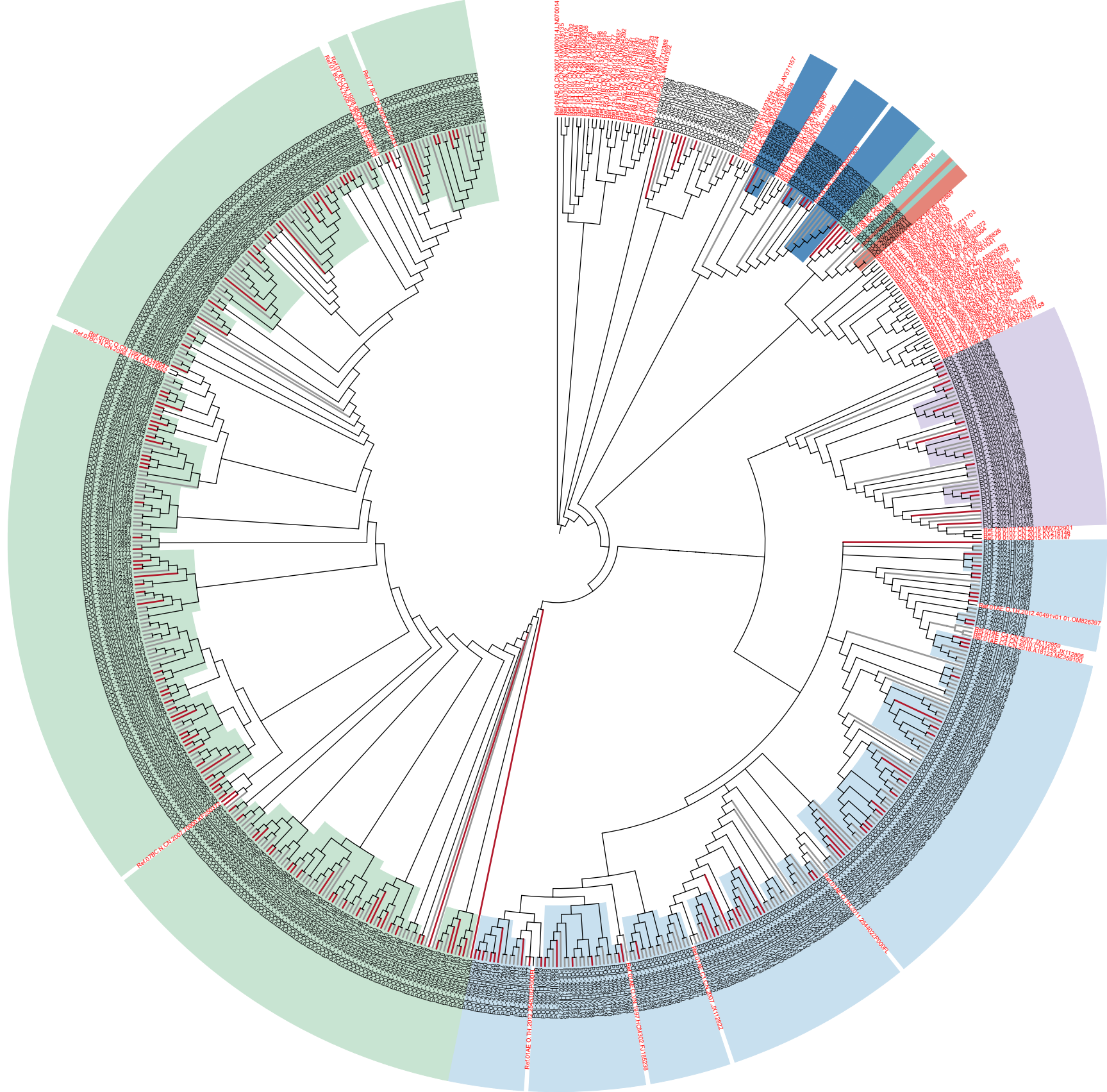

0.40

Supplement: Supplementary file 1 [file viruses-16-01174-s001.zip › Figure S3.pdf]

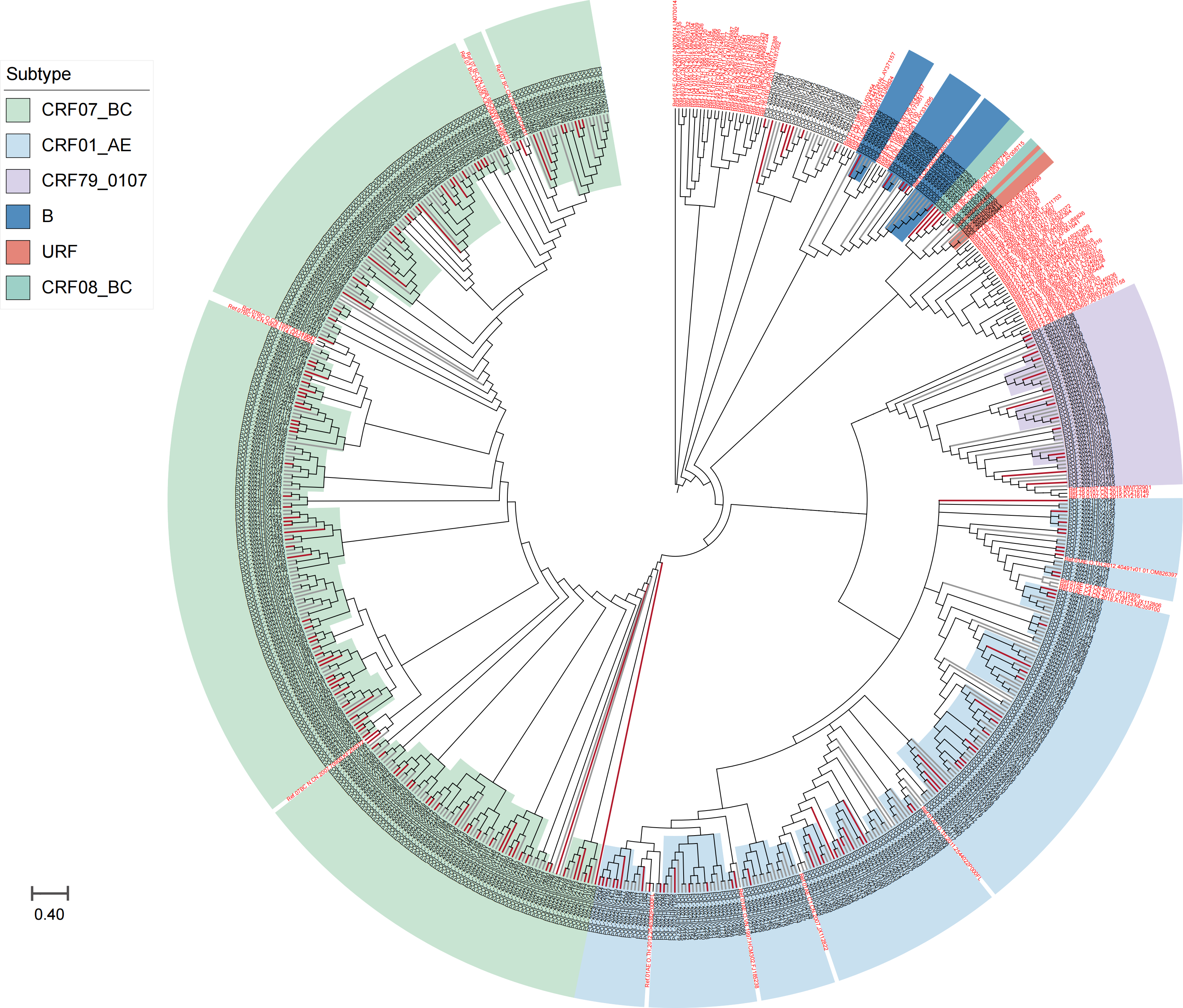

Supplement: Supplementary file 1 [file viruses-16-01174-s001.zip › Figure S3.png]

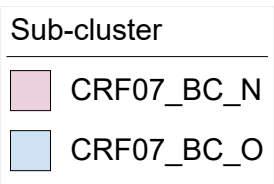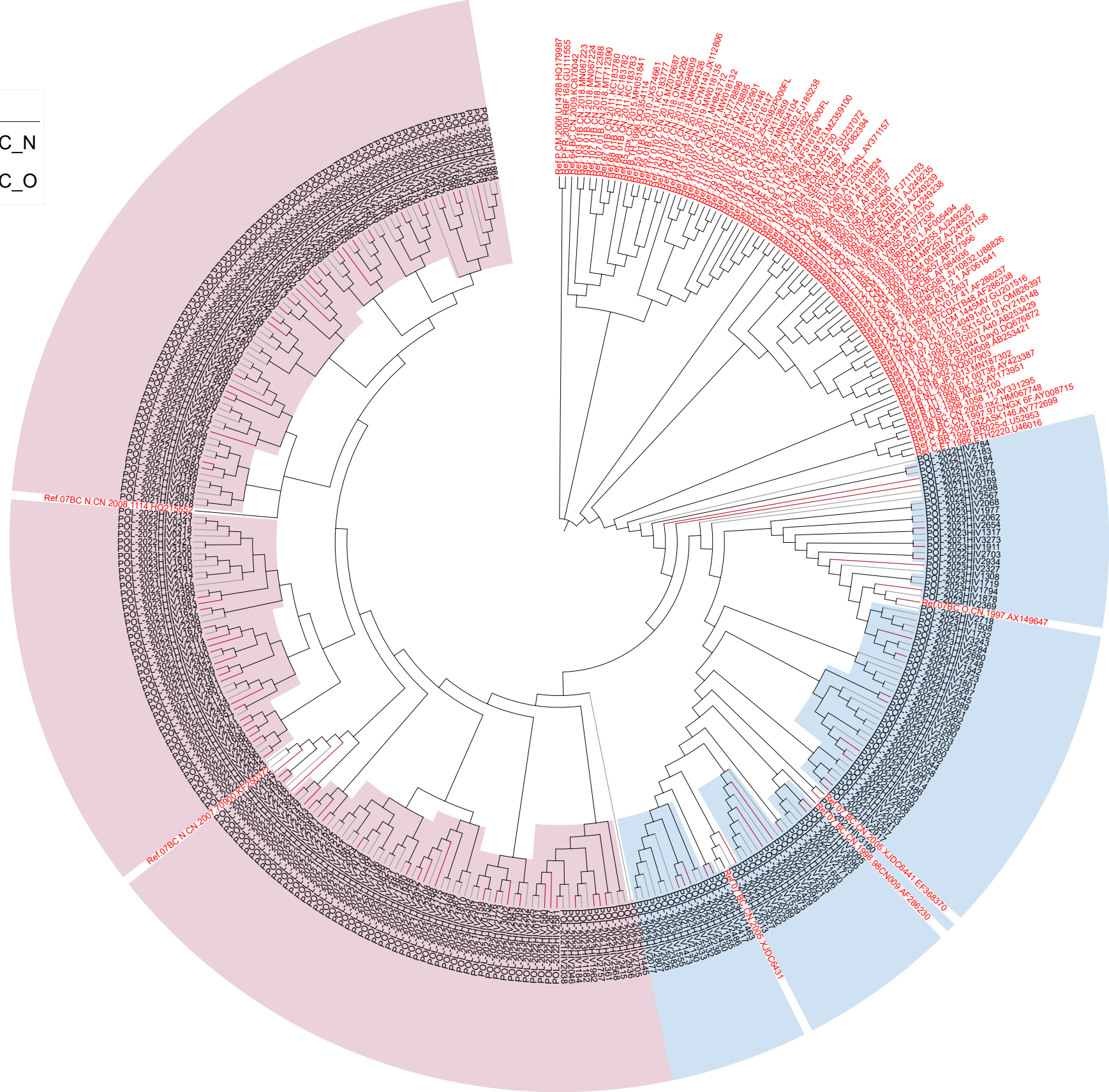

Supplement: Supplementary file 1 [file viruses-16-01174-s001.zip › Supplementary Figure S1.pdf]

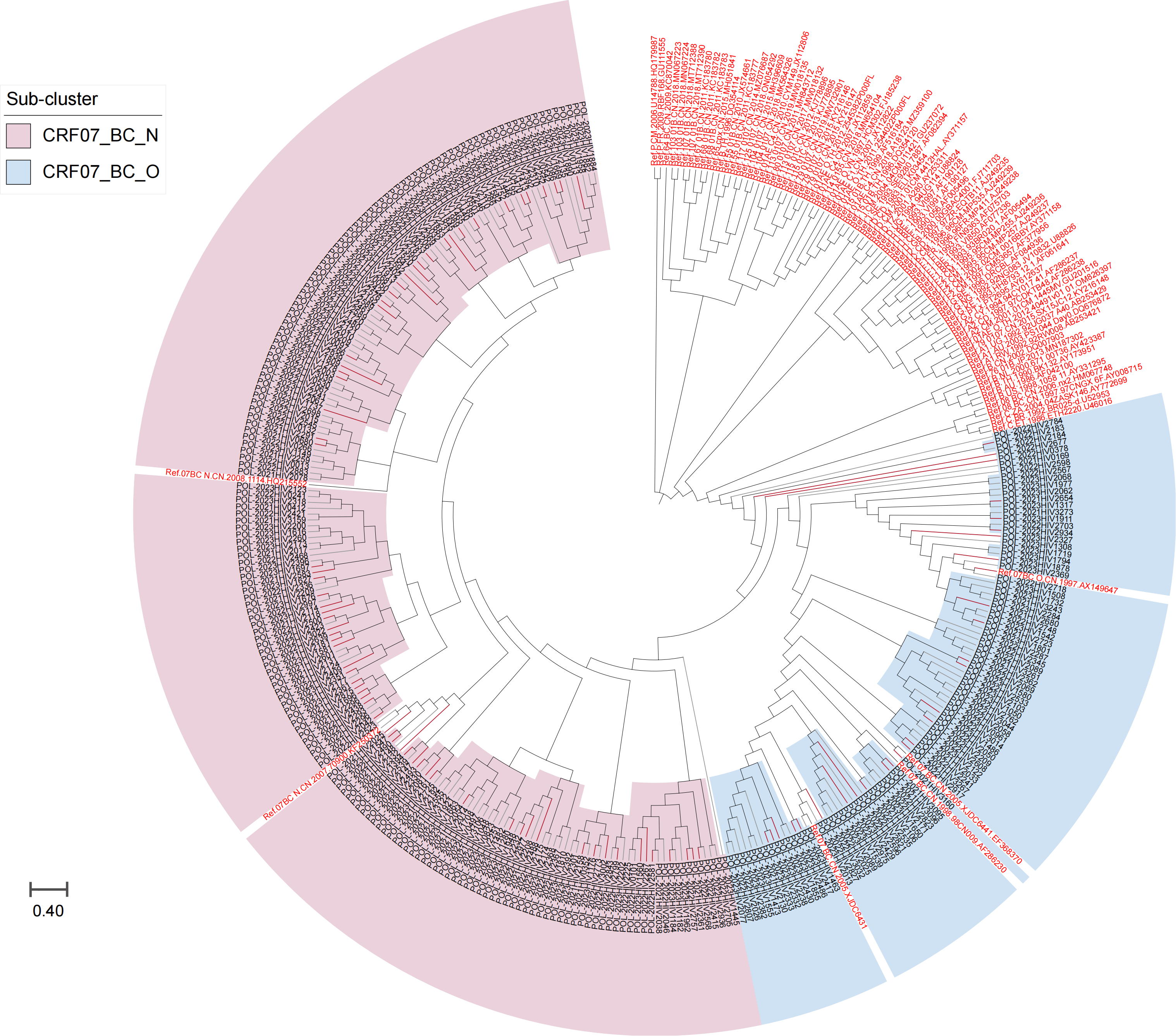

Supplement: Supplementary file 1 [file viruses-16-01174-s001.zip › Supplementary Figure S1.png]

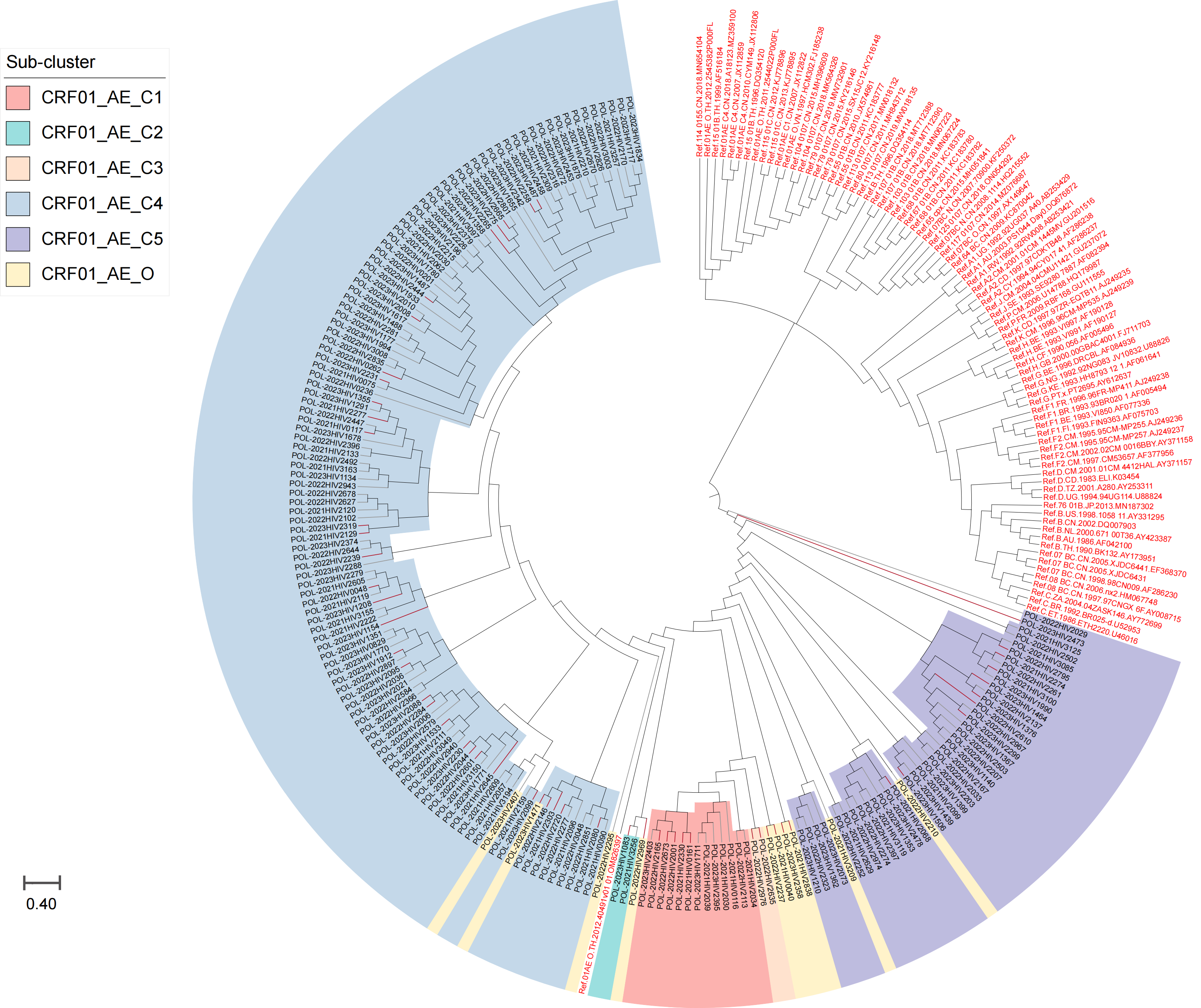

Supplement: Supplementary file 1 [file viruses-16-01174-s001.zip › Supplementary Figure S2.png]
