# Supplementary material for: Characteristics of Subtype and Molecular Transmission Networks among Newly Diagnosed HIV-1 Infections in Patients Residing in Taiyuan City, Shanxi Province, China, from 2021 to 2023
Source: Viruses. 2024 Jul 22;16(7):1174. doi: 10.3390/v16071174 (PMC11281631; doi:10.3390/v16071174)
Supplement: Supplementary file 1 [file Supplementary Figure S2.pdf › Supplementary Figure S2.pdf]

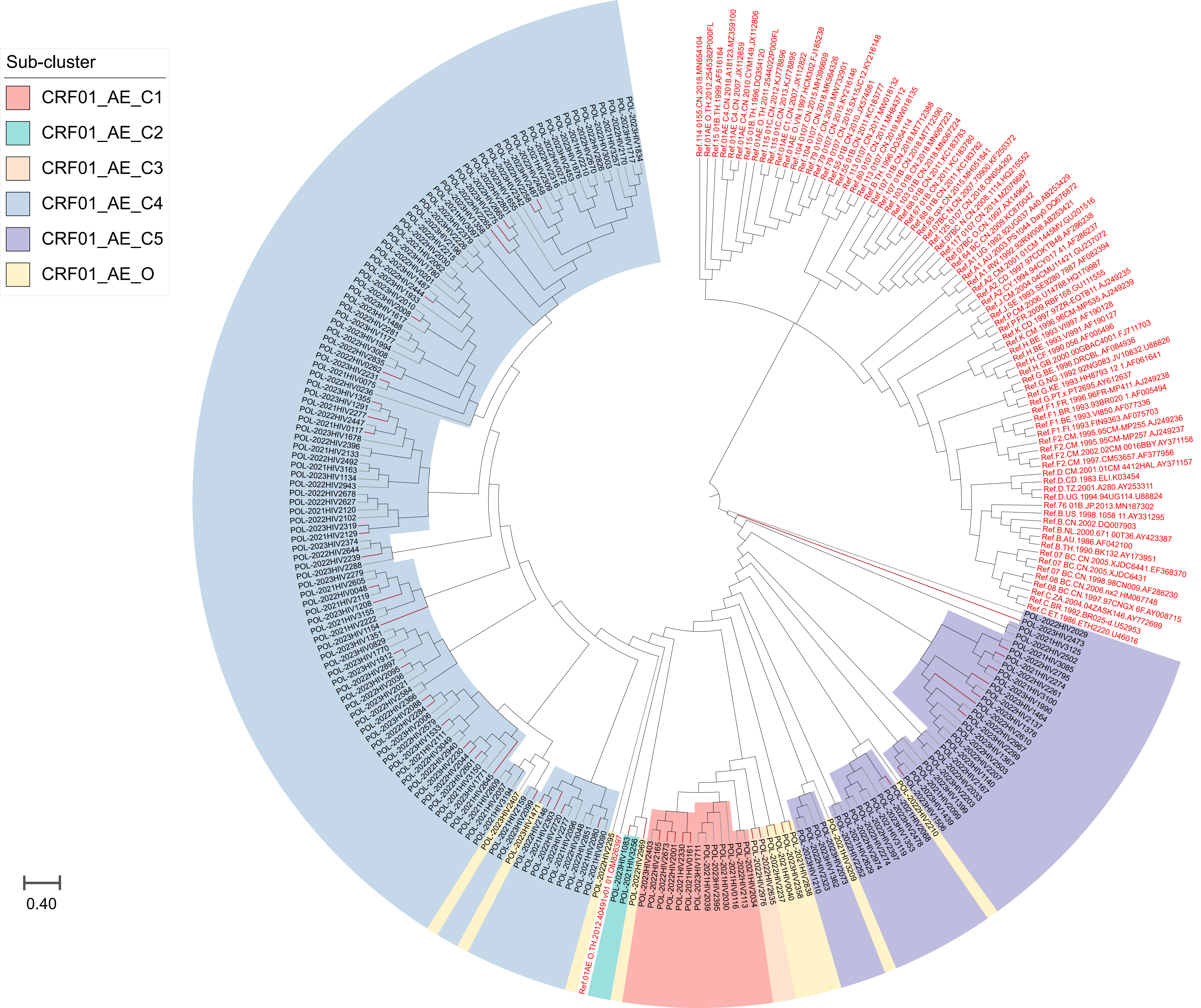
Sub-cluster
CRF01_AE_C1
CRF01_AE_C2
CRF01_AE_C3
CRF01_AE_C4
CRF01_AE_C5
CRF01_AE_O
0.40
